# The prognostic significance of incomplete hematological recovery in pediatric patients with low/intermediate risk AML and negative MRD after induction 1

**DOI:** 10.3389/fonc.2025.1661036

**Published:** 2025-10-28

**Authors:** Nessma M. Shahin, Mahmoud Hammad, Hanafy Hafez, Sherine Salem, Nahla El-Sharkawy, Amr Elnashar, Leslie Lehmann, Alaa El-Haddad

**Affiliations:** ^1^ Department of Pediatric Oncology, Children Cancer Hospital (57357), Cairo, Egypt; ^2^ Department of Pediatric Oncology, National Cancer Institute, Cairo University, Cairo, Egypt; ^3^ Department of Clinical Pathology, Children Cancer Hospital (57357), Cairo, Egypt; ^4^ Department of Clinical Pathology, National Cancer Institute, Cairo University, Cairo, Egypt; ^5^ Department of Clinical Research, Children Cancer Hospital (57357), Cairo, Egypt; ^6^ Pediatric Oncology, Harvard Medical School, Boston Children’s Hospital, Dana-Farber Cancer Institute, Boston, MA, United States

**Keywords:** pediatric acute myeloid leukemia (AML), minimal residual disease (MRD), complete remission (CR), incomplete remission (CRi), morphological remission

## Abstract

**Background:**

Pediatric acute myeloid leukemia (AML) accounts for approximately 25% of childhood hematologic malignancies. Outcomes have markedly improved, especially in low-risk and intermediate-risk patients, with overall survival (OS) rates approaching 80–85%. Prognosis is primarily determined by cytogenetic/molecular risk and minimal residual disease (MRD) status following induction therapy. While complete remission (CR) traditionally requires morphologic clearance of leukemia with full hematologic recovery, some patients achieve morphologic remission with incomplete recovery (CRi). Although adult studies associate CRi with poor prognosis, its relevance in MRD-negative pediatric AML remains unclear. This study evaluates the prognostic significance of hematologic recovery in MRD-negative, low/intermediate-risk pediatric AML.

**Methods:**

We conducted a retrospective analysis of 120 pediatric AML patients treated at CCHE-57357 between 2012 and 2020 who achieved MRD negativity (<0.1% by 8–10 color flow cytometry) after Induction I. Risk stratification followed WHO/ELN guidelines. For exploratory purposes, intermediate-risk patients with MRD <0.1% were reclassified as “MRD-defined low risk.” Patients were categorized by hematologic recovery: CR (ANC ≥1000/µL, platelets ≥100,000/µL), partial hematological recovery (CRh) (ANC ≥500/µL and/or platelets ≥50,000/µL), and CRi (ANC 500/µL and/or platelets <50,000/µL). Outcomes included OS, relapse-free survival (RFS), and event-free survival (EFS).

**Results:**

Among 120 patients (median age 8.5 years), 25 (21%) achieved CR, 17 (14.3%) CRh, and 78 (64.7%) CRi. CRi patients had numerically lower 5-year OS (63.3%) compared to CRh (76%) and CR (71.8%), though differences were not statistically significant. Platelet recovery alone (complete Platelet recovery (CRp) vs incomplete platelet recovery (CRip) showed a trend toward prognostic relevance (5-year OS: 73.3% vs 57.1%), also non-significant. Infectious complications were common: six sepsis-related deaths occurred in the low-risk group and four in the standard-risk group, with ICU admissions disproportionately higher in standard-risk patients (12 vs 1). CRi patients experienced longer hospital stays and required more transfusion support.

**Conclusion:**

In MRD-negative pediatric AML, incomplete hematologic recovery did not significantly predict inferior survival, though trends suggest potential prognostic value—particularly in low-risk patients. CRi may reflect treatment-related toxicity or infectious complications rather than residual disease. These findings support a more nuanced interpretation of remission depth and highlight the need for larger, multi-institutional studies incorporating molecular risk refinement and clinical context.

## Introduction

Acute myeloid leukemia (AML) accounts for approximately one-quarter of childhood hematological malignancies and remains a major therapeutic challenge due to its biological heterogeneity. Although survival outcomes have improved substantially in recent decades reaching 80–85% in favorable-risk groups relapse continues to represent the leading cause of treatment failure and mortality in pediatric AML (1–3).

The primary objective of induction therapy is to achieve complete remission (CR), defined by <5% marrow blasts on morphology, negative minimal residual disease (MRD), and concurrent recovery of peripheral counts, specifically an absolute neutrophil count (ANC) ≥1000/µL and platelet count ≥100,000/µL. However, many patients who enter morphologic remission fail to fully normalize counts. Such patients are classified as having incomplete hematologic recovery (CRi: ANC ≤ 500/µL and/or platelets ≤ 50,000/µL) or partial recovery (CRh: ANC ≥500/µL and/or platelets ≥50,000/µL). Platelet recovery has also been studied separately, with CRp defined as platelets ≥50,000/µL and CRip as ≤ 50,000/µL (4).

The prognostic relevance of these distinctions is not yet fully established in pediatrics. Many adults studies demonstrated that CRi and CRp often correlate with MRD positivity and are associated with increased relapse risk, independent of MRD status (5). In pediatrics, however, the impact of hematologic recovery on relapse risk is less clear, particularly in low- and intermediate-risk patients, since high-risk patients typically proceed to allogeneic transplantation in first remission (6).

The prognostic value of MRD negativity has been increasingly recognized, yet its interplay with hematologic recovery is not fully understood. To address this, the European LeukemiaNet (ELN) updated response criteria in 2022, formally incorporating MRD into remission categories (CR-MRD neg, CRh-MRD neg, CRi-MRD neg), thereby providing a more refined assessment of post-induction disease status (7, 8).

Given these considerations, it remains critical to investigate whether incomplete hematologic recovery retains prognostic significance in pediatric AML patients who achieve MRD negativity. We hypothesized that peripheral count recovery and MRD clearance represent complementary but distinct measures of leukemia eradication. The present study therefore aimed to evaluate the prognostic implications of hematologic recovery in low- and intermediate-risk pediatric AML patients achieving MRD negativity following Induction I therapy.

## Patients and methodology

This retrospective study was conducted at the Children’s Cancer Hospital Egypt (CCHE-57357) and included 120 pediatric patients (≤18 years) diagnosed with *de novo* acute myeloid leukemia (AML) between January 2012 and December 2020. Patients with acute promyelocytic leukemia (APL), Down syndrome, myelodysplastic syndrome (MDS), therapy-related AML (t-AML), myeloid sarcoma, Fanconi anemia, or those who died before the end-of-induction response assessment were excluded. Eligible patients were classified as low- or intermediate-risk AML according to the World Health Organization (WHO) criteria and had achieved minimal residual disease (MRD)-negative status (<0.1% by 8–10 color flow cytometry) following Induction I. The minimum follow-up period was two years after completion of therapy. Written informed consent was obtained from patients or guardians before diagnostic workup or treatment initiation, and the study protocol was approved by the Institutional Review Board (IRB).

Baseline disease assessments included morphology, immunophenotyping, cytogenetics, and molecular analyses. Post-Induction I, bone marrow aspiration was performed on day 21, and MRD was assessed by multiparameter flow cytometry (MFC) using 8–10 color monoclonal antibody panels, using the Leukemia-Associated Immunophenotype (LAIP) method in accordance with European LeukemiaNet (ELN) consensus recommendations (9).

### Treatment protocol

All patients were treated according to the CCHE-57357 AML protocol, adapted from sequential Children’s Oncology Group (COG) trials: AAML0531 (2007–2014), AAML1031 (2014–2021), and AAML1831 (2020–present). (NCT01371981) (10) While the backbone of therapy remained consistent—comprising standard induction and consolidation phases—risk-adapted modifications were introduced over time, particularly in AAML1031 and AAML1831, which emphasized MRD-guided intensification. Treatment protocol All patients were treated according to the CCHE-57357 AML protocol, adapted from sequential Children’s Oncology Group (COG) trials: AAML0531 (2007–2014), AAML1031 (2014–2021), and AAML1831 (2020–present). (NCT01371981) (10) While the backbone of therapy remained consistent—comprising standard induction and consolidation phases—risk-adapted modifications were introduced over time, particularly in AAML1031 and AAML1831, which emphasized MRD-guided intensification. Shown in **Table 1**.

**Table 1:** Overview of treatment protocols used at CCHE 57357 for pediatric AML patients.

Protocol	Years active	Source protocol	Key features	Gemtuzumab use
CCHE 57357 AML	2007 - 2014	COG AAML0531	Standard induction + consolidation	Not included
CCHE 57357 AML	2014 - 2021	COG AAML1031	Risk-adapted therapy; MRD-guided decisions	Not included
CCHE 57357 AML	2020 - Present	COG AAML1831	Incorporates targeted agents; MRD-driven	Not yet implemented

### Risk stratification

- Low Risk (LR): Patients with favorable cytogenetics, including core-binding factor (CBF) abnormalities [t(8;21)(q22;q22.1); RUNX1::RUNX1T1, inv(16)(p13.1;q22) or t(16;16)(p13.1;q22); CBFB::MYH11], NPM1 mutation, or biallelic CEBPA mutations.
- Intermediate Risk (IR): Patients lacking both favorable and adverse cytogenetic markers.
- High Risk (HR): Patients with adverse cytogenetics such as monosomy 7, monosomy 5, complex karyotypes (>3 abnormalities), or FLT3-ITD with a high allelic ratio (>0.4).

Following Induction I, intermediate-risk patients were further stratified by MRD results. Patients with MRD <0.1% were categorized as “MRD-defined low risk” for exploratory purposes, though they were analyzed separately from cytogenetically low-risk patients.

### Definitions of response and hematologic recovery

Patients who achieved morphologic remission and MRD negativity by MFC (<0.1%) on day 21 after the first induction, were evaluated for peripheral count recovery on day 28 post-induction I. The absolute neutrophil count (ANC) and platelet count were used to categorize patients into three groups (11):

- Complete remission (CR): <5% marrow blasts, MRD <0.1%, platelet count ≥100,000/µL, and ANC ≥1000/µL.
- CR with partial recovery (CRh): Morphologic remission and MRD <0.1%, with platelet count ≥50,000/µL and/or ANC ≥500/µL, but not fulfilling CR criteria.
- CR with incomplete recovery (CRi): Morphologic remission and MRD <0.1%, but with platelet count <50,000/µL and/or ANC <500/µL.
- Complete platelet recovery (CRp): Platelet count ≥50,000/µL.
- Incomplete platelet recovery (CRip): Platelet count <50,000/µL.
- Refractory disease: Persistence of ≥5% marrow blasts after Induction II.
- Relapse: Reappearance of leukemic blasts in peripheral blood or ≥5% blasts in bone marrow after initial CR.

## Statistical analysis

The researchers used software (SPSS version 20) to analyze data. This study used both descriptive and inferential statistics to analyze the data. For categorical variables, in each category the prevalence of participants and the percentage of the total sample they represent are presented. For numerical variables, the results are summarized by either the average (mean) and its variability (standard deviation) or the middle value (median) and the range of values around it (interquartile range), depending on whether the data is normally distributed.

This method was employed to describe the baseline characteristics of the study participants, the outcomes of interest, and any other relevant factors that were taken into account during the analysis. Patient survival was reported using Kaplan-Meier curves and results compared between groups using a “log-rank test.”

The primary endpoint was RFS, which is the time from the date of complete remission to the date of relapse or mortality. OS was the secondary endpoint, defined as the time from the date of diagnosis to the date of mortality or last contact. EFS was defined as the duration from the date of diagnosis to the occurrence of an event, either relapse, refractory disease, or mortality.

## Results

Following Induction I, all 120 patients with low- or intermediate-risk AML achieved MRD negativity by multiparameter flow cytometry. The median age at diagnosis was 8.5 years (range, 0–17 years), and the male-to-female ratio was 1.6:1. The median follow-up duration was 60.7 months (range, 1.1–112.5 months). Risk distribution was balanced, with 52.5% classified as low risk and 47.5% as intermediate risk. Baseline characteristics are detailed in **Table 2**.

**Table 2:** Baseline patients characteristics.

Characteristic	Value
Age (years)	Median (IQR): 8.5 (3–12) Range: 0–17
Initial TLC (×10^9^/L)	Median (IQR): 20 (8.2–62.7) Range: 2–370
Gender	Male: 74 (61.7%) Female: 46 (38.3%)
Karyotype	<46: 8 (6.8%) =46: 94 (79.7%) >46: 18 (13.5%)
Initial Risk	Low Risk (LR): 63 (52.5%) Intermediate Risk (IR): 57 (47.5%)

**Figure 1:**
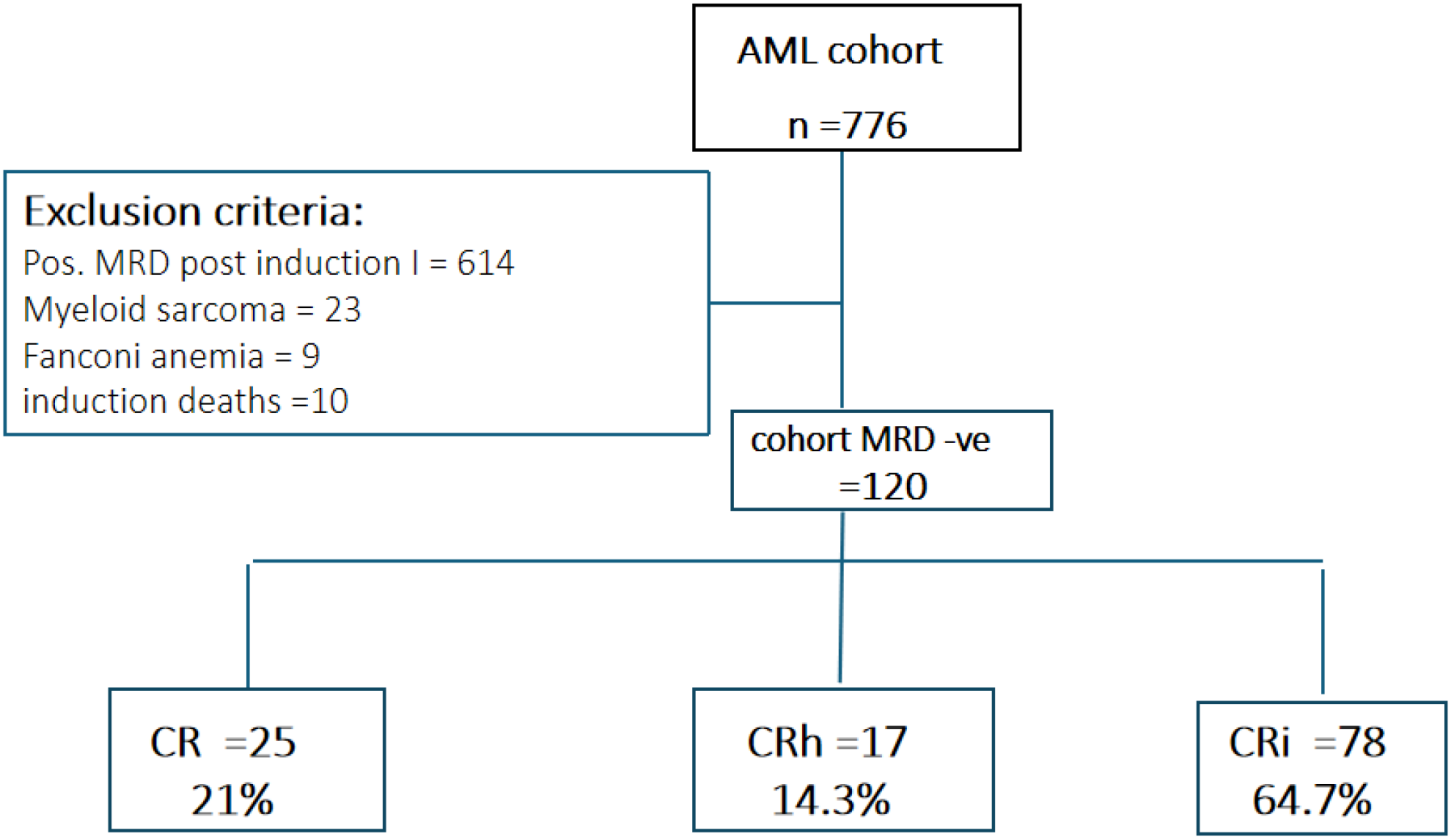
CONSORT diagram describing cohort.

In terms of hematologic recovery, 25 patients (21%) achieved complete remission (CR), 17 (14.3%) achieved partial recovery (CRh), and 78 (64.7%) had incomplete recovery (CRi). No significant associations were observed between recovery category and baseline demographic or disease-related characteristics (**Table 3**, **Figure 1**).

**Table 3:** Association between hematological recovery and initial disease characteristics among pediatric AML patients.

Characteristic	CR (n = 25)	CRh (n = 17)	CRi (n = 78)	*p*-value
Age (years)	Median (IQR): 7.5 (—) Range: 0–17	9 (—) 1–17	9 (—) 1–15	0.3
Initial TLC (×10^9^/L) Total leukocyte count	Median (IQR): 28 Range: 2–370	18 Range: 2–163	15.5 Range: 2–300	0.7
Karyotype	<46: 2 (8.0%) =46: 20 (80.0%) >46: 3 (12.0%)	<46: 2 (11.8%) =46: 10 (58.8%) >46: 5 (29.4%)	<46: 4 (5.1%) =46: 70 (89.7%) >46: 4 (5.1%)	0.6
Initial Risk	Low Risk: 15 (60.0%) Intermediate Risk: 10 (40.0%)	Low Risk: 7 (41.2%) Intermediate Risk: 11 (58.8%)	Low Risk: 38 (48.7%) Intermediate Risk: 40 (51.3%)	0.2

The predominance of CRi in this cohort suggests that incomplete hematologic recovery may frequently reflect treatment-related marrow suppression or supportive care complications rather than persistent leukemia, underscoring the importance of integrating MRD status and clinical context when interpreting remission depth.

### Survival outcomes

For the entire cohort, the estimated 5-year overall survival (OS), relapse-free survival (RFS), and event-free survival (EFS) were 66.2% ± 4 (95% CI: 57.6–74.7%), 73.4% ± 4 (95% CI: 64.8–82.0%), and 62.9% ± 4 (95% CI: 54.1–71.6%), respectively. Relapse occurred in 27 patients, the majority of whom (20/27) had failed to achieve complete hematologic recovery following Induction I. Similarly, of the 40 deaths recorded, 28 occurred in patients who did not achieve CR, indicating a numerical disadvantage associated with incomplete recovery.

When outcomes were stratified by remission category, patients with CRi consistently demonstrated lower survival compared with those achieving CR or CRh, although differences did not reach statistical significance, likely reflecting the limited sample size. Specifically, 5-year OS was 63.3% ± 5 (95% CI: 52.5–74.0%) for CRi, 76.0% ± 10 (95% CI: 55.0–96.5%) for CRh, and 71.8% ± 9 (95% CI: 54.0–89.5%) for CR (p = 0.5). Corresponding 5-year EFS was 59.5% ± 5 (95% CI: 48.4–70.5%) for CRi, 69.7% ± 11 (95% CI: 47.4–91.5%) for CRh, and 71.8% ± 9 (95% CI: 54.0–89.5%) for CR (p = 0.4). RFS rates followed a similar pattern: 69.5% ± 5 (95% CI: 58.3–80.6%) for CRi, 79.0% ± 10 (95% CI: 57.7–100%) for CRh, and 81.6% ± 8 (95% CI: 65.8–97.9%) for CR (p = 0.4).

**Figure 2:**
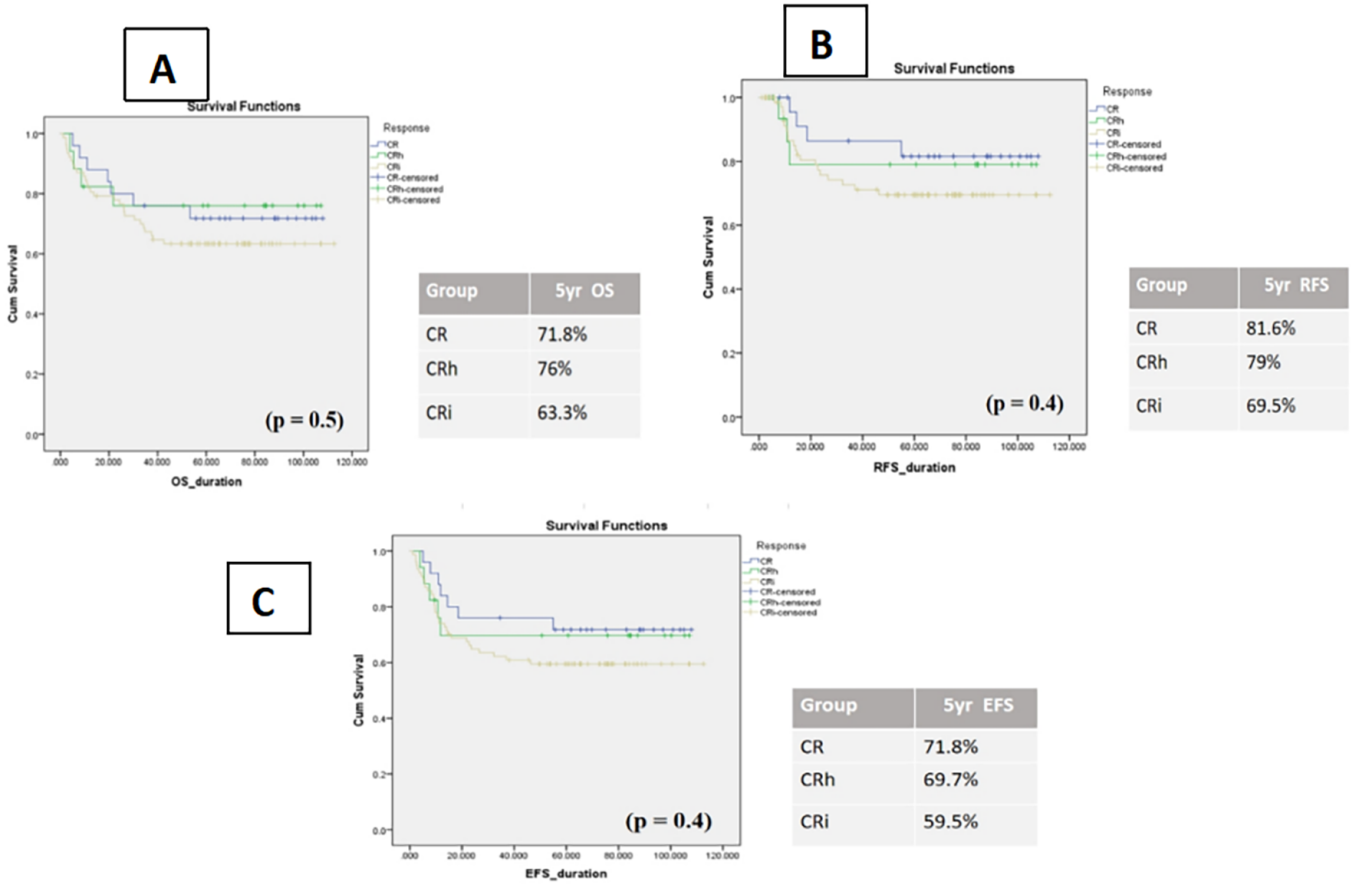
Kaplan–Meier curves of impact of count recovery on survival in three subgroups **(A)** OS patients, **(B)** relapse-free survival, **(C)** event-free survival.

Although not statistically significant, these consistent trends suggest that incomplete hematologic recovery may be associated with inferior long-term outcomes, reinforcing the need for larger studies to clarify its prognostic role (**Figure 2**).

**Figure 3:**
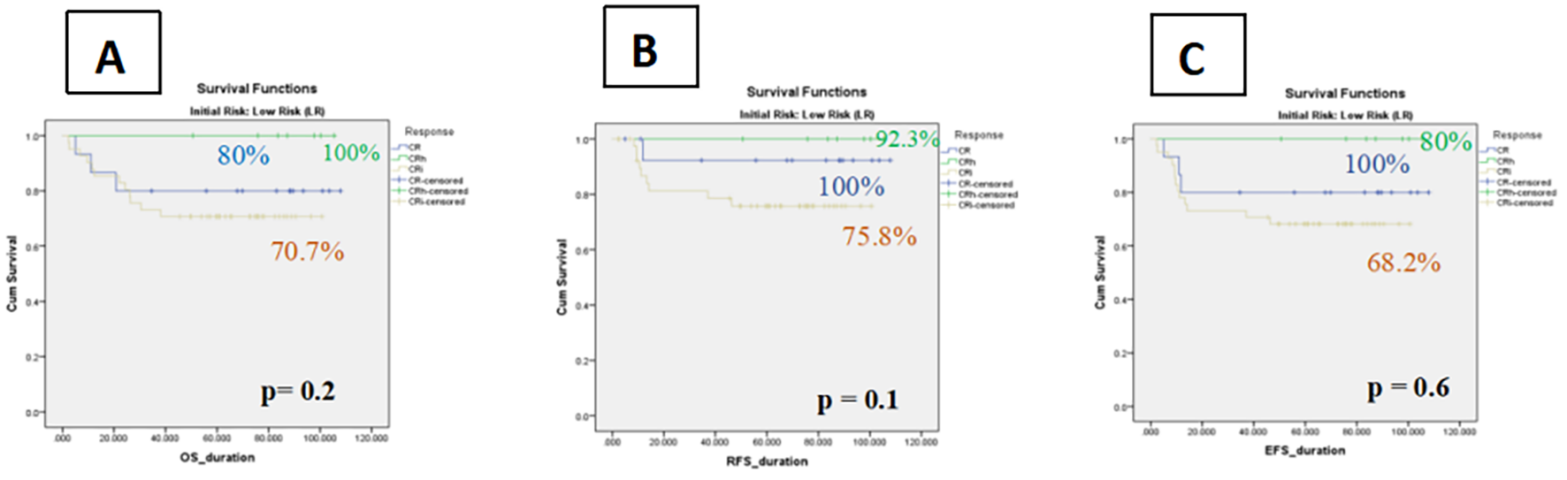
Kaplan–Meier curves of impact of count recovery on survival among Low Risk groups **(A)** The OS in three subgroups, **(B)** Relapse-free survival rates of patients in three subgroups, **(C)** The event-free survival of patients in three subgroup.

Within the low-risk cohort, patients achieving CRi demonstrated numerically inferior outcomes compared with those achieving CR or CRh. The 5-year OS was 70.7% ± 7 (95% CI: 58.8–84.6%) for CRi versus 80.0% ± 10 (95% CI: 59.7–100%) for CR and 100% for CRh. This disadvantage was primarily driven by higher relapse rates in the CRi group, reflected by a 5-year RFS of 75.8% ± 7 (95% CI: 61.9–89.5%) compared with 92.3% ± 7 (95% CI: 77.8–100%) for CR and 100% for CRh. Similarly, the 5-year EFS was 68.2% ± 7 (95% CI: 53.9–82.4%) for CRi, compared with 80.0% ± 10 (95% CI: 59.7–100%) for CR and 100% for CRh. Despite these clear numerical differences, none of the comparisons reached statistical significance (**Figure 3**).

A subgroup analysis of patients with core binding factor (CBF) AML further highlighted this trend: CRi patients had a higher relapse risk compared with those in CR/CRh (45.6% vs. 22%).

**Figure 4:**
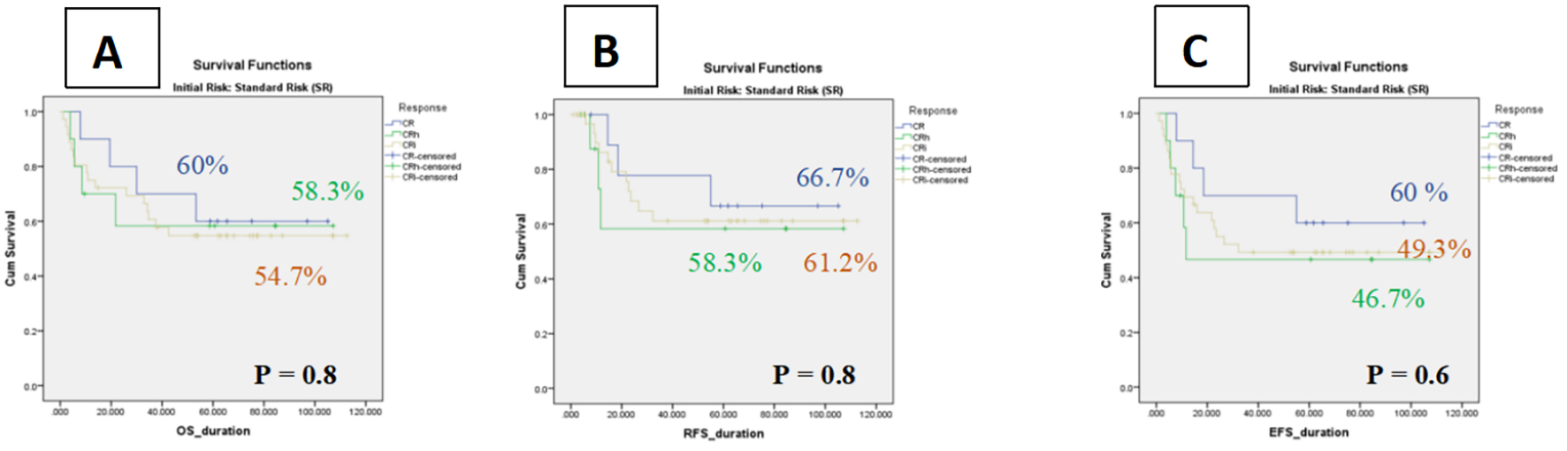
Kaplan–Meier curves of impact of count recovery on survival among Intermediate Risk groups **(A)** The OS in three subgroups, **(B)** Relapse-free survival rates of patients in three subgroups, **(C)** The event-free survival of patients in three subgroup.

In contrast, among intermediate-risk patients, hematologic recovery status did not significantly affect outcomes. The 5-year OS was 54.7% ± 8 (95% CI: 38.2–71.2%) for CRi, 58.3% ± 16 (95% CI: 26.7–89.8%) for CRh, and 60.0% ± 15 (95% CI: 29.6–90.3%) for CR. Corresponding RFS values were 61.2% ± 9 (95% CI: 43.1–79.1%) for CRi, 58.3% ± 18 (95% CI: 21.9–94.7%) for CRh, and 66.7% ± 15 (95% CI: 35.8–97.4%) for CR. Similarly, 5-year EFS was 49.3% ± 8 (95% CI: 32.7–65.7%) for CRi, 46.7% ± 16 (95% CI: 14.1–79.1%) for CRh, and 60.0% ± 15 (95% CI: 29.6–90.3%) for CR. These findings indicate that, within the intermediate-risk group, the depth of hematologic recovery (CR, CRh, or CRi) did not meaningfully influence survival or event-free outcomes (**Figure 4** **).**

Relapse-free survival (RFS) varied across remission categories, though none of the differences reached statistical significance. Among patients achieving CR, 21 of 25 (84%) remained relapse-free, compared with 57 of 77 (74%) in the CRi group (p = 0.3). Outcomes for CRh were nearly identical to CR, with 14 of 17 patients (82%) relapse-free (p = 0.88 vs. CR). When comparing CRh to CRi, 82% versus 74% of patients remained relapse-free, respectively (p = 0.47).

Taken together, both CR and CRh groups showed slightly higher proportions of patients maintaining remission compared with CRi, but these differences did not achieve statistical significance (**Table 4**). The consistent numerical disadvantage in CRi suggests a possible trend toward inferior RFS, though the study was underpowered to confirm this association.

**Table 4:** Impact of hematological recovery on relapse and outcome among entire cohort.

Comparison	Response groups	RFS no (n, %)	RFS yes (n, %)	Total (n)	*p*-value
CR vs. CRi	CR (n=25)	21 (84.0%)	4 (16.0%)	25	0.30
	CRi (n=77)	57 (74.0%)	20 (26.0%)	77	
	Total	78	24	102	
CR vs. CRh	CR (n=25)	21 (84.0%)	4 (16.0%)	25	0.88
	CRh (n=17)	14 (82.4%)	3 (17.6%)	17	
	Total	35	7	42	
CRh vs. CRi	CRh (n=17)	14 (82.4%)	3 (17.6%)	17	0.47
	CRi (n=77)	57 (74.0%)	20 (26.0%)	77	
	Total	71	23	94	

### Impact of platelet recovery on outcomes in the cohort

Platelet recovery following Induction I appeared to influence clinical outcomes across both low-risk (LR) and intermediate-risk (IR) AML patients. Patients who failed to achieve platelet counts ≥50,000/µL (CRip) consistently demonstrated inferior survival compared with those attaining complete platelet recovery (CRp).

**Figure 5:**
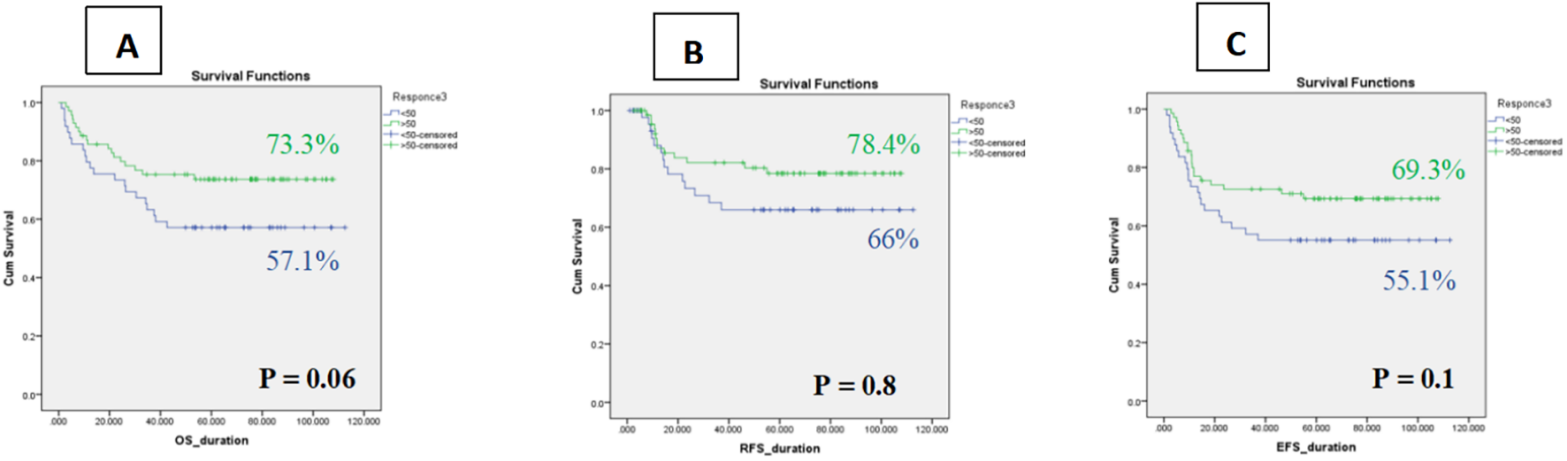
Kaplan–Meier curves of Impact of platelet recovery on survival among whole cohort **(A)** OS, **(B)** RFS, **(C)** EFS.

In the combined LR/IR cohort, the 5-year OS for CRip patients was 57.1% ± 7 (95% CI: 43.2–70.9%) versus 73.3% ± 5 (95% CI: 63.2–84.1%) for CRp. Similarly, 5-year RFS was 66.0% ± 7 (95% CI: 51.4–80.4%) for CRip compared with 78.4% ± 5 (95% CI: 67.9–88.8%) for CRp. Event-free survival showed the same pattern, with 55.1% ± 7 (95% CI: 41.1–69.0%) in CRip versus 69.3% ± 5 (95% CI: 58.3–80.2%) in CRp. Although not statistically significant, these findings suggest that delayed platelet recovery may be associated with worse long-term outcomes, highlighting its potential prognostic relevance within MRD-negative pediatric AML (**Figure 5**).

When analyzed by risk group, the association between inadequate platelet recovery and inferior outcomes persisted. In the low-risk cohort, patients with incomplete platelet recovery (CRip) demonstrated lower survival rates compared with those achieving platelet recovery (CRp). The 5-year OS, EFS, and RFS for CRip were 66.7% ± 9 (95% CI: 47.8–85.5%), 66.7% ± 9 (95% CI: 47.8–85.5%), and 76.7% ± 9 (95% CI: 58.2–94.4%), respectively, versus 82.1% ± 6 (95% CI: 70.0–94.0%), 79.3% ± 6 (95% CI: 66.5–92.0%), and 86.1% ± 5 (95% CI: 74.7–97.4%) for CRp (p = 0.1, 0.3, and 0.2, respectively).

A similar but more pronounced trend was observed in the intermediate-risk cohort. CRip patients had a 5-year OS of 48.0% ± 10 (95% CI: 28.4–67.5%), EFS of 44.0% ± 9 (95% CI: 24.5–63.4%), and RFS of 55.0% ± 11 (95% CI: 33.1–76.8%), compared with 62.9% ± 8 (95% CI: 45.4–80.4%), 56.4% ± 9 (95% CI: 38.4–74.3%), and 67.3% ± 9 (95% CI: 48.6–86.0%) for CRp (p = 0.2, 0.1, and 0.3, respectively) (**Table 5**).

**Table 5:** Impact of platelet recovery on survival among low risk & intermediate risk group.

Low risk	OS	EFS	RFS
CRip	66.7%	66.7%	76.7%
CRp	82.1%	79.3%	86.1%
	p=0.1	p=0.3	p=0.2

Intermediate risk	OS	EFS	RFS
CRip	48%	44%	55%
CRp	62.9%	56.4%	67.3%
	p=0.2	p=0.1	p=0.3

Although these differences did not reach statistical significance, the consistent numerical disadvantage for CRip suggests that inadequate platelet recovery may reflect weaker hematopoietic recovery or greater treatment-related toxicity, potentially contributing to higher relapse risk. This observation aligns with our discussion on the interplay between delayed count recovery, infectious complications, and transfusion dependence, underscoring the importance of supportive care in interpreting remission depth.

Notably, Infectious complications were common: six sepsis-related deaths occurred in the low-risk group and four in the standard-risk group, with ICU admissions disproportionately higher in standard-risk patients (12 vs 1). CRi patients experienced longer hospital stays and required more transfusion support.

## Discussion

For children with acute myeloid leukemia (AML), the primary therapeutic goal is to achieve complete remission (CR), which has long been associated with improved long-term survival. Traditionally, CR has required both morphologic remission (<5% bone marrow blasts) and recovery of peripheral blood counts. However, a subset of patients achieve morphologic clearance without full hematologic recovery, termed CR with incomplete recovery (CRi). While CRi is recognized as an adverse prognostic factor in adult AML, its significance in pediatric patients remains less clearly defined (12, 13).

Incomplete count recovery may reflect several processes, including persistent leukemic disease at levels below detection, chemotherapy-related myelosuppression, or complications such as infection. The integration of measurable residual disease (MRD) assessment into response evaluation has refined prognostic stratification. Patients achieving both hematologic recovery and MRD negativity (CR MRD-neg) have the most favorable outcomes, which underpins the updated 2022 European LeukemiaNet (ELN) response criteria (8). Nevertheless, patients with CRi or CRp may harbor residual disease or face treatment-related vulnerabilities that could adversely affect outcomes (5, 14, 15).

Evidence from adult AML strongly supports the prognostic value of hematologic recovery. In a large multicohort analysis of 7,235 patients, Appelbaum et al. reported inferior survival in both CRi and CRh compared with CR, with CRi associated with a 49% increased risk of mortality. Our findings align partially with those reported by this previous study that demonstrated inferior survival outcomes in patients with CRi compared to those achieving full hematologic recovery. However, their analysis did not incorporate MRD status, a key prognostic marker in pediatric AML. In contrast, our cohort was uniformly MRD-negative post-Induction I, which may explain the absence of statistically significant survival differences between CRi and CR groups. This suggests that MRD negativity may mitigate the adverse prognostic impact of incomplete hematologic recovery in pediatric populations—a hypothesis that warrants further investigation. Moreover, while Appelbaum et al. relied solely on cytogenetic and molecular risk stratification, our study incorporated MRD-based refinement of risk groups. This distinction highlights the evolving role of MRD in pediatric AML and underscores the need for integrated models that combine genetic, immunophenotypic, and treatment response data (4).

Similarly, CIBMTR analyses and other adult cohorts demonstrated that both MRD positivity and incomplete recovery independently predicted poorer outcomes. Our findings contrast with those of Percival et al., who reported inferior post-transplant outcomes in adult AML patients with CRi—even among those who were MRD-negative prior to allo-HSCT. While both studies used a consistent MRD cutoff (<0.1%) via multiparameter flow cytometry, our pediatric cohort did not undergo transplant and was evaluated earlier in the treatment course. This distinction may explain the absence of statistically significant survival differences in our MRD-negative CRi group (16, 17). Moreover, Percival et al. applied ELN-based risk stratification, similar to our approach. Their results underscore the prognostic weight of remission depth even in MRD-negative patients, suggesting that CRi may reflect underlying disease biology or marrow vulnerability. In pediatric AML, however, our data suggest that MRD negativity may offset the adverse impact of incomplete hematologic recovery—at least in the early phases of therapy (16).

While our study focused on pediatric AML patients with low/intermediate risk and MRD negativity following Induction I, the implications of incomplete hematologic recovery (CRi) extend into post-remission strategies, particularly allogeneic hematopoietic stem cell transplantation (allo-HSCT). Percival et al. demonstrated that adult AML patients undergoing allo-HSCT in first remission had significantly worse outcomes when transplanted in CRi compared to full CR, even among those who were MRD-negative (16). This suggests that CRi may reflect underlying marrow vulnerability or residual disease biology not captured by MRD alone. In contrast, our pediatric cohort—treated without transplant—did not show statistically significant survival differences between CRi and CR groups, possibly due to earlier treatment phase, age-related marrow resilience, or the mitigating effect of MRD negativity. These findings collectively highlight the need for tailored transplant timing and remission depth criteria, especially in MRD-negative patients, and support further investigation into whether CRi should influence transplant decisions in pediatric AML.

Platelet recovery specifically has been linked to prognosis: Çiftçiler et al. showed that late platelet recovery was associated with higher mortality and relapse risk in adults. Collectively, these studies confirm that in adults, depth of hematologic recovery meaningfully influences survival even in MRD-negative patients (15, 18). In contrast, our findings in pediatric low- and intermediate-risk AML patients achieving MRD negativity after Induction I suggest a more limited prognostic role for hematologic recovery. Across our cohort, patients with CRi or CRh demonstrated numerically lower survival compared with CR, but differences in 5-year OS, RFS, and EFS did not reach statistical significance. Notably, CRi patients accounted for the majority of relapses and deaths, and incomplete platelet recovery was consistently associated with inferior numerical outcomes. These findings suggest a trend toward poorer prognosis with CRi, though our study may have been underpowered to detect statistically significant effects.

Subgroup analyses supported this interpretation. In the low-risk cohort, CRi was associated with lower OS (70.7% vs. 80.0% for CR and 100% for CRh) and RFS (75.8% vs. 92.3% for CR and 100% for CRh). In contrast, no meaningful differences were observed among intermediate-risk patients, indicating that the prognostic value of hematologic recovery may be attenuated in higher-risk groups where disease biology and transplant decisions exert stronger influences on outcome.

The prognostic relevance of platelet recovery also remains uncertain. While our cohort demonstrated numerically lower OS, RFS, and EFS in patients with incomplete platelet recovery, these differences were not significant. This parallels adult data, yet highlights that in pediatrics, the effect may be more modest or confounded by supportive care factors such as infection burden and transfusion dependence (19).

Infectious complications emerged as a notable factor that may have influenced hematologic recovery and survival outcomes across risk groups. In the low-risk (LR) cohort, six patients died due to sepsis and one required intensive care unit (ICU) admission, while in the standard-risk (SR) group, four patients succumbed to sepsis and twelve required ICU-level care. These events likely reflect episodes of febrile neutropenia, bloodstream infections, and culture-positive sepsis, which are known to prolong marrow suppression and delay count recovery. The disproportionate burden of ICU admissions in the SR group may have contributed to the higher incidence of incomplete hematologic recovery (CRi) observed in this cohort. Moreover, the presence of severe infections during the recovery window could confound the interpretation of CRi as a prognostic marker, as delayed count recovery may be driven by transient inflammatory or infectious stress rather than residual disease. These findings underscore the importance of accounting for infectious morbidity when evaluating remission depth and support the need for integrated models that distinguish biologically driven CRi from treatment-related or infection-associated cytopenias.

In addition to infectious morbidity, cardiac compromise emerged as a critical complication influencing treatment outcomes. Within the standard-risk (SR) group, two patients developed cardiac impairment secondary to severe infectious episodes and died during therapy. These cases likely reflect sepsis-associated myocardial dysfunction or drug-induced cardiotoxicity exacerbated by systemic infection, as described in prior literature including the study by Rubnitz et al. (20), which emphasized the vulnerability of pediatric AML patients to organ dysfunction during intensive chemotherapy. The presence of cardiac compromise not only contributed directly to mortality but may have indirectly delayed hematologic recovery, further complicating remission assessment.

These findings underscore the need to interpret incomplete count recovery (CRi) within the broader context of treatment-related toxicity and systemic complications, rather than as a sole surrogate for residual disease. Incorporating clinical events such as cardiac dysfunction into prognostic models may improve risk stratification and guide supportive care strategies in future pediatric AML protocols.

Importantly, outcomes in pediatric AML are generally superior to those in adults, and adult prognostic markers may not translate directly. In the largest available U.S. pediatric dataset, Pommert et al. similarly found no significant correlation between hematologic recovery and survival in patients treated on recent COG trials, though they observed a trend toward lower DFS in CRi patients (12). Together with our results, these findings support the argument that pediatric-specific response criteria are needed, rather than extrapolating directly from adult AML.

Notably, none of the patients in this cohort received gemtuzumab ozogamicin, despite its incorporation into AAML0531 for favorable-risk patients. This exclusion was due to limited drug availability and regulatory constraints during the study period. As such, while treatment regimens were broadly standardized, the absence of gemtuzumab may have influenced outcomes in specific subgroups, particularly those with core-binding factor AML. Future cohorts treated under AAML1831 may reflect more contemporary therapeutic strategies, including targeted agents and immunoconjugates.

Using older risk assessment methods without incorporating Next-Generation Sequencing (NGS) has significant limitations, as it can lead to misclassification of patients and potentially suboptimal treatment decisions.

In summary, while incomplete hematologic recovery (particularly CRi) was not an independent predictor of outcome in our MRD-negative pediatric cohort, the consistent trends toward inferior survival and higher relapse risk highlight that hematologic recovery may still carry prognostic relevance. Larger, multicenter studies with molecularly refined stratification and standardized supportive care reporting will be essential to clarify whether CRi identifies a subset of pediatric patients who may benefit from modified therapeutic approaches.

## Study limitations

This study has several limitations that warrant consideration. First, the retrospective, single-center design may introduce selection bias and limit generalizability. Second, the relatively small sample size restricted statistical power, particularly for subgroup analyses, and may explain why observed trends did not achieve significance. Third, Older risk stratification systems often fail to identify high-risk patients who appear to have a more favorable prognosis based on traditional methods. A patient with a normal karyotype, for example, would be classified as intermediate-risk by older standards. However, NGS can reveal a hidden high-risk mutation, like TP53 or RUNX1, that significantly worsens their prognosis and would warrant more intensive therapy, such as an allogeneic stem cell transplant. Without NGS, these patients may receive standard chemotherapy and have a much higher risk of relapse. Fourth, assessment of hematologic recovery was performed at fixed time points, which may not fully capture delayed count recovery. Finally, supportive care variables including infection burden, ICU admissions, and transfusion dependence likely influenced outcomes in CRi patients, confounding interpretation of hematologic recovery as an isolated prognostic factor.

## Clinical implications

Our findings suggest that in pediatric AML patients who achieve MRD negativity, incomplete hematologic recovery may reflect treatment-related toxicity, infection burden, or delayed marrow regeneration rather than persistent leukemia. While not independently predictive of survival in this cohort, the consistent numerical disadvantage observed in CRi and CRip patients highlights the need for careful monitoring, aggressive infection control, and optimization of transfusion support in this subgroup. Importantly, the lack of statistical significance underscores that adult-derived prognostic definitions may not directly apply to children, reinforcing the importance of developing pediatric-specific response criteria that integrate both MRD and hematologic recovery. Finally, in the context of allogeneic hematopoietic cell transplantation, our data suggest that CRi alone should not automatically trigger transplant referral in MRD-negative patients, and that remission depth must be interpreted alongside clinical status, toxicity profile, and supportive care needs. These refinements could improve risk-adapted therapy and supportive care strategies, ultimately enhancing outcomes in pediatric AML.

## Conclusion and future directions

Consistent with our results, Pommert et al., in the largest U.S. pediatric AML cohort from recent COG trials (AAML0531, AAML1831), reported no statistically significant association between hematologic recovery and survival, though a trend toward inferior disease-free survival was observed in CRi patients. They concluded that adult-derived response criteria should not be applied directly to pediatric AML, underscoring the need for pediatric-specific definitions of treatment response.

In our cohort of low- and intermediate-risk pediatric patients who achieved MRD negativity after Induction I, hematologic recovery status (CRi vs. CR/CRh) was not significantly associated with OS, RFS, or EFS. Nevertheless, the consistent trend toward higher relapse risk among CRi patients mirroring observations in COG datasets suggests that complete hematologic recovery may represent an optimal endpoint, even in the context of MRD negativity.

Taken together, these findings highlight the importance of further large-scale, prospective pediatric studies to clarify whether CRi reflects a biologically distinct subgroup within MRD-negative AML. Such research will be critical to determine whether patients with incomplete recovery require treatment intensification or alternative supportive strategies. Ultimately, these data, alongside other pediatric evidence, reinforce the need for response criteria tailored specifically to children, integrating both MRD and hematologic recovery into risk stratification and therapeutic decision-making.

### Key takeaway

In MRD-negative pediatric AML, incomplete hematologic recovery (CRi) was not an independent predictor of survival but consistently correlated with higher relapse risk, underscoring the need for pediatric-specific response criteria and validation in larger prospective cohorts.

## Data Availability

The original contributions presented in the study are included in the article/supplementary material. Further inquiries can be directed to the corresponding authors.
